# Ceramides in tracheal aspirates of preterm infants: Marker for bronchopulmonary dysplasia

**DOI:** 10.1371/journal.pone.0185969

**Published:** 2018-01-18

**Authors:** Esther van Mastrigt, Salomé Zweekhorst, Bas Bol, Jeroen Tibboel, Joost van Rosmalen, Janneke N. Samsom, André A. Kroon, Johan C. de Jongste, Irwin K. M. Reiss, Martin Post, Mariëlle W. Pijnenburg

**Affiliations:** 1 Division of Pediatric Pulmonology, Erasmus MC–Sophia Children’s Hospital, Rotterdam, the Netherlands; 2 Division of Neonatology, Erasmus MC–Sophia Children’s Hospital, Rotterdam, the Netherlands; 3 Program of Physiology and Experimental Medicine, Hospital for Sick Children, Toronto, ON, Canada; 4 Department of Biostatistics, Erasmus MC, Rotterdam, The Netherlands; 5 Laboratory of Pediatrics, Erasmus MC, Rotterdam, the Netherlands; University of Giessen Lung Center, GERMANY

## Abstract

**Background:**

In an experimental mouse model we showed that ceramides play a role in the pathogenesis of bronchopulmonary dysplasia (BPD) and are a potential target for therapeutic intervention. We investigated whether ceramides are detectable in tracheal aspirates (TAs) of preterm infants and differ between infants with or without BPD.

**Methods:**

Infants born ≤ 32 weeks of gestational age in need of mechanical ventilation in the first week of life were included. TAs were obtained directly after intubation and at day 1, 3, 5, 7, and 14. Ceramide concentrations were measured by tandem mass spectrometry. At 36 weeks postmenstrual age BPD was defined as having had ≥ 28 days supplemental oxygen.

**Results:**

122 infants were included, of which 14 died and 41 developed BPD. All infants showed an increase in ceramides after the first day of intubation. The ceramide profile differed significantly between preterm infants who did and did not develop BPD. However, the ceramide profile had no additional predictive value for BPD development over GA at birth, birth weight and total days of mechanical ventilation.

**Conclusions:**

Ceramides are measurable in TAs of preterm born infants and may be an early marker for BPD development.

## Introduction

Bronchopulmonary dysplasia (BPD) is a serious complication affecting preterm born infants, with an incidence ranging from 4% in infants born at a gestational age (GA) of 31 weeks to 56% in infants born at a GA of < 26 weeks [1]. BPD is defined as need for oxygen supplementation for ≥ 28 days at 36 weeks postmenstrual age [2]. Genetic predisposition and exposure to environmental factors, such as chorioamnionitis, preeclampsia, GA at birth, birth weight, mechanical ventilation, supplemental oxygen exposure, postnatal infections, persistent ductus arteriosus (PDA) and malnutrition have been implicated in the aetiology of BPD. To date, treatment is symptomatic and children with BPD are at increased risk of long term respiratory sequelae [3].

Sphingolipids are important structure-bearing constituents of the cell membrane that also function as regulatory molecules in cell proliferation and cell death, endothelial barrier function, angiogenesis, and immune response [4–6]. Altered sphingolipid levels have been shown to play a role in asthma [7–9], cystic fibrosis [10,11], and cigarette-smoke or radiation induced lung injury [12–14]. This stresses the biological importance of the sphingolipid metabolism in lung disease [5]. Two important sphingolipids are ceramides and sphingosine-1-phosphate (S1P). Ceramides act as a precursor for all other sphingolipids; S1P is generated from ceramide via sphingosine [15]. Ceramides and S1P play an important role in apoptosis, with ceramides stimulating apoptosis and cell cycle arrest and S1P stimulating cell survival and proliferation [16]. Apoptosis may be critical in BPD as increased apoptosis has been found in pulmonary epithelial cells of BPD patients and in animal models of BPD [17,18]. Recently we observed a transient increase in ceramide concentrations in bronchoalveolar lavage (BAL) fluid of a mouse model of BPD during the first 2–4 weeks of hyperoxia. Supplementation of D-sphingosine, a synthetic precursor of S1P, during normoxic recovery of hyperoxia-induced lung damage accelerated normalization of ceramide concentrations and improved the hyperoxia-induced alveolar arrest in this mouse model [19]. In addition, Kunzmann *et al*. found that intra-amniotic exposure to LPS, which induced antenatal chorioamnionitis, significantly increased ceramide concentrations in the lungs of fetal sheep [20]. These findings suggest that ceramides are involved in the development of BPD, may be used as an early biomarker for BPD, and might be a target for new therapeutic interventions.

The aim of the present study was to translate these findings to humans, by analysing ceramide profiles in tracheal aspirates (TAs) of preterm infants. We hypothesized that ceramide concentrations in TAs were higher in preterm born infants who developed BPD compared to those who did not, and may be an early biomarker for BPD.

## Methods

### Study subjects and design

Preterm infants (≤32 weeks GA) in need of mechanical ventilation in the first week of life were recruited at the Neonatal Intensive Care Unit of Erasmus MC-Sophia Children’s Hospital. Children with a congenital disorder affecting lung structure and/or function and children who were intubated only to administer surfactant were excluded. We obtained TAs directly after intubation, and as long as children were intubated at consecutive days 1, 3, 5, 7 and 14, during routine suctioning procedures. We included children from whom we were able to obtain the first tracheal aspirate directly after birth or at day 1. BPD was diagnosed according to criteria of Jobe and Bancalari [2]. BPD severity was assessed by means of an oxygen reduction test at 36 weeks postmenstrual age [21]. Relevant maternal and neonatal characteristics including maternal age, preeclampsia, chorioamnionitis, antenatal steroids, mode of delivery, GA at birth, birth weight, sex, surfactant treatment, early or late onset sepsis, persistent ductus arteriosus (PDA), postnatal systemic steroids, days on mechanical ventilation and days on oxygen were prospectively collected from patients records. Written informed consent was obtained from the parents. The study was approved by the medical ethical committee of Erasmus MC, Rotterdam, The Netherlands (MEC-2013-062, NL43229.078.13).

### Tracheal aspirate collection and processing

TAs were collected directly after intubation, and at days 1, 3, 5, 7, and 14 as long as the patient was intubated. TAs were obtained at least 6h after surfactant administration. During the procedure 0.5 ml of sterile isotonic saline was instilled into the endotracheal tube, and after 5 sec suctioning was performed with a 5F catheter positioned at the end of the endotracheal tube. TAs were directly stored at 4°C. After centrifugation for 5 min at 300×g, the supernatant was collected, aliquoted in 1.5 ml extra low binding Eppendorf tubes (BiozymTC, Landgraaf, the Netherlands), and stored at -80°C until analysis. Subsequently, cell suspensions were stored in 100 μL 1x phosphate-buffered saline (PBS). Cells were counted and a maximum of 4 standardized cytospins with 50,000 cells per slide were prepared. Cytospin preparations were made with a cytocentrifuge (Cytospin 4, Thermo Scientific, Breda, the Netherlands) at 680rpm for 7min with low acceleration. The cytospins were air dried for at least 24h and subsequently wrapped in aluminium foil and stored at -20°C. For each TA sample one cytospin was stained with May-Grünwald Giemsa staining. Per slide a differential cell count was performed by two observers (EM and IK) [22,23].

### Liquid chromatography tandem mass spectrometry

Ceramide concentrations (ng/mL) in TAs were measured as previously described [19]. Briefly, lipids were extracted from TA samples (200μL) and ceramides were separated using high performance liquid chromatography (LC) and quantified by tandem mass spectrometry (MS/MS). The analyses were performed at the Analytical Facility for Bioactive Molecules of the Hospital for Sick Children, Toronto, ON, Canada.

### Sample size calculation

The sample size calculation was based on the results of a previously performed mouse study [19]. The mean variance in sphingolipid concentrations within this mouse model was 0.6. The incidence of BPD was estimated to be 21% [24]. By including 150 children in total, and thus an expected number of 30 patients with BPD, we calculated that we would be able to detect a 30% difference in ceramide concentrations between children with and without BPD with a power of 0.8. We believed this expected difference to be realistic, because the mean difference in ceramide concentrations between mice exposed to normooxia and hyperoxia was 1.3.

### Statistical analysis

Continuous variables were expressed as mean ± standard deviation (SD) for normally distributed data or median (interquartile range, IQR) for data that were not normally distributed, and categorical variables were expressed as n (%). We compared infants who did not develop BPD with those who did develop BPD or died because of respiratory failure before 36 weeks PMA using Fisher’s exact test for categorical variables or the Mann-Whitney U test for continuous variables. Ceramide concentrations were compared for each time point separately using the Mann-Whitney U test. Ceramide concentrations below the detection limit were given the value of the lower detection limit (0.03 ng/ml). The concentrations of each individual ceramide were above the detection limit in the majority of TAs, except for CerDiHy 18:1, of which 92.1% of the samples had concentrations below the detection limit. Therefore, this ceramide was excluded from further analysis. Ceramide concentrations were logarithmically transformed in order to obtain an approximately normal distribution. Spearman’s rho was used to assess the correlation between individual ceramides and the correlation between ceramides and cell counts. Cell counts between different time points were compared using the Kruskal-Wallis test.

We performed a principal component analysis for all time points together and included the logarithmically transformed data of all individual ceramides. This analysis revealed that the information could be captured in a single component explaining 84.2% of the variance in the data. Because the factor loadings of the principal component analysis showed that this component was highly similar to the mean of the logarithmically transformed ceramides, we computed a new variable representing the mean of the logarithmically transformed ceramides per patient and per time point.

Linear mixed models were used to examine the change over time of the mean logarithmically transformed ceramide concentrations, providing estimates of the average change in the samples. These models account for the repeated measures structure of the data and can handle missing values in the dependent variable [25]. Independent variables in the linear mixed effect models were BPD outcome, GA at birth, birth weight SDS, time between birth and intubation and total days of invasive ventilation at 36 weeks PMA, and time at which TAs were obtained. Time at which TAs were obtained (post intubation day 0, 1, 3, 5, 7 and 14) was treated as a categorical variable with day 0 as reference level. A random intercept was included in the linear mixed models to account for within-subject correlations.

To explore whether longitudinal observations of the ceramide profile or individual ceramides predict the development of BPD we used a two-step approach. First, a linear regression of the log-transformed ceramides on time, coded as continuous variable, for each patient and ceramide was performed. The estimates of the intercept and the slope of the ceramide profile and individual ceramides were then included as independent variables in a logistic regression for the development of BPD. Patients for whom ceramide concentrations were not available at two or more time points were excluded from this analysis, because no linear regression could be performed in that case. GA at birth, birth weight SDS, time between birth and intubation and total days of invasive ventilation were included as independent variables in this logistic regression model. The calibration of the logistic regression model was assessed using the Hosmer-Lemeshow test. All statistical tests used a two-sided significance level of 0.05, but a Bonferroni correction for multiple comparisons was applied in analyses that were performed for each time point separately, leading to an adjusted significance level of 0.01. SPSS version 21.0 software (SPSS, Inc., Chicago, IL) and Excel version 2010 software were used for statistical analyses (syntax available S2 Data).

## Results

### Patient characteristics

**Fig 1:**
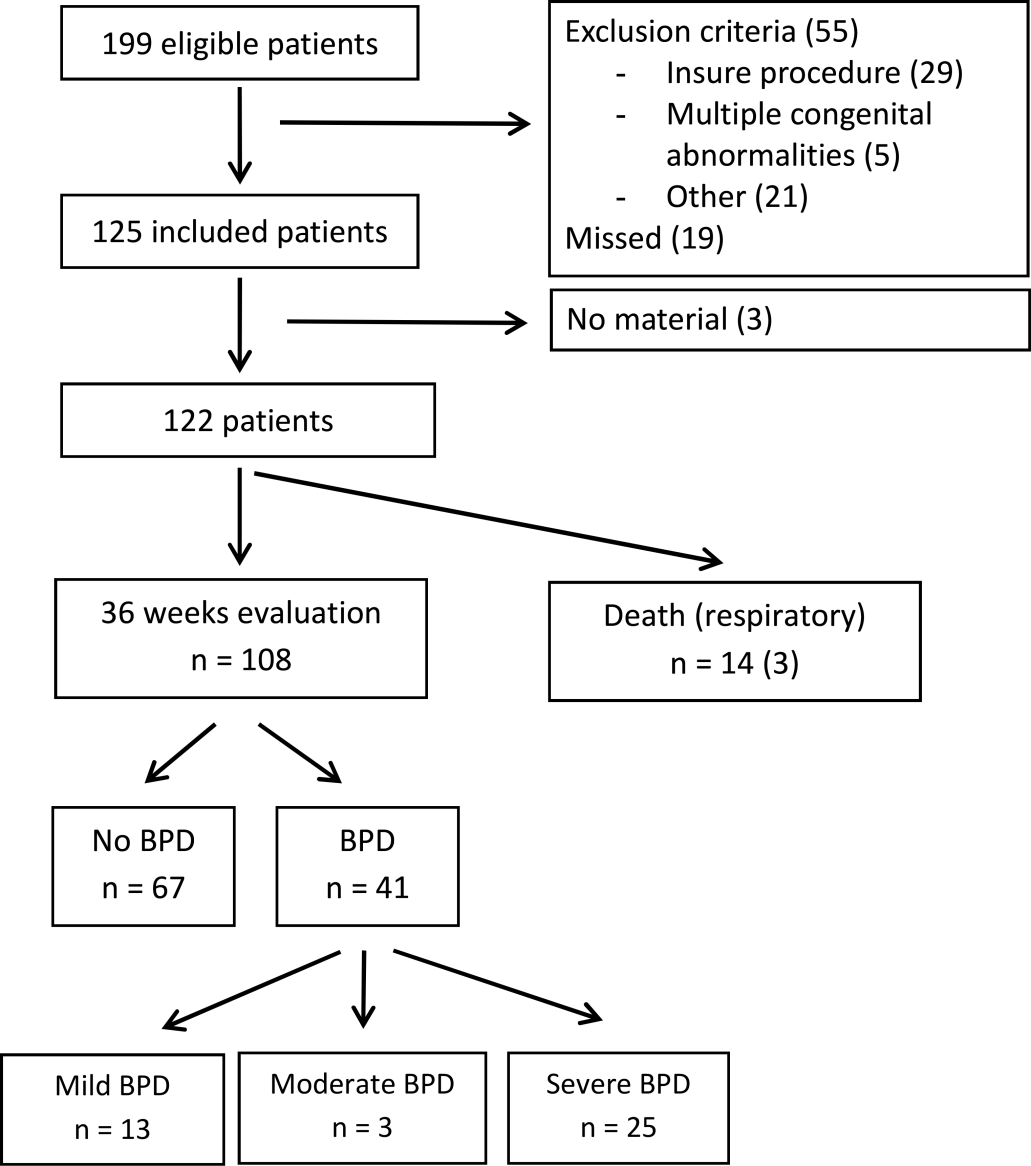
Flow diagram study population. Overview of the eligible and included patients between September 2013 and December 2014.

We included 122 preterm born infants with a median GA (IQR) of 27.2 (25.6–29.6) weeks and a mean birth weight (SD) of 1003 (371) grams between September 2013 and December 2014 (Table 1). Fourteen infants died before 36 weeks PMA, of whom 3 due to respiratory failure. Forty-one infants (33.6%) developed BPD, 16 mild-moderate and 25 severe (Fig 1). Infants who developed BPD were born at significantly lower GA, with lower birth weight, received more antenatal corticosteroids and postnatal surfactant, experienced more complications like late onset sepsis and PDA, and had a significantly longer duration of mechanical ventilation and oxygen supplementation.

**Table 1:** Patient characteristics.

	No BPD	BPD or respiratory death	*p* value
**Number of patients**	67	44	
**Number of TAs**	Day 0: 51 Day 1: 48 Day 3: 18 Day 5: 12 Day 7: 8 Day 14: 3	Day 0: 34 Day 1: 39 Day 3: 34 Day 5: 35 Day 7: 31 Day 14: 29	
**Gestational age (weeks)**	29.4 (27.9–30.4)	25.9 (24.5–26.3)	< 0.001
**Birth weight (gram)**	1205 ± 346	768 ± 230	< 0.001
**Sex (male: female)**	33: 34	25: 19	0.446
**Antenatal corticosteroids**	No: 17 (25.4%) Not complete: 17 (25.4%) Yes, complete: 33 (49.2%)	No: 3 (6.8%) Not complete: 12 (17.9%) Yes, complete: 29 (65.9%)	< 0.001
**Maternal preeclampsia**	14 (20.9%)	11 (25.0%)	0.647
**Chorioamnionitis**	14 (20.9%)	15 (34.1%)	0.203
**Postnatal surfactant**	No: 12 (17.9%) 1 dose: 36 (53.7%) ≥ 2 doses: 19 (28.4%)	No: 4 (9.1%) 1 dose: 12 (27.3%) ≥ 2 doses: 28 (63.6%)	< 0.001
**Early onset sepsis**	2 (3.0%)	2 (4.5%)	0.648
**Late onset sepsis**	14 (20.9%)	23 (52.3%)	0.001
**PDA**	24 (35.8%)	38 (86.4%)	< 0.001
**Total days mechanical ventilation**	2 (1–6)	22 (11–33)	< 0.001
**Total days supplemental oxygen exposure**	3 (0–13)	55 (40–67)	< 0.001

Definition of abbreviations: BPD; bronchopulmonary dysplasia, PDA; persistent ductus arteriosus. Maternal preeclampsia was defined as newly developed hypertension (systolic ≥ 140 mmHg or diastolic ≥ 90 mmHg) after 20 weeks of pregnancy and proteinuria (protein-creatinine ratio ≥ 30 mg/mmol or ≥ 300 mg/24u). Chorioamnionitis was determined based on placental pathology findings. Treatment with prenatal corticosteroids was considered adequate when infants were born at least 12 hours after the second antenatal corticosteroid administration. Early onset sepsis was defined as a positive blood culture in the first 72 hours after birth, late onset sepsis as a positive blood culture after the first 72 hours after birth and in the first 3 months of life. Continuous variables are expressed as mean ± standard deviation (SD) for normally distributed data or median (interquartile range, IQR) for data that were not normally distributed. Categorical data are expressed as n (%).

### Ceramides in tracheal aspirates of preterm born infants

**Fig 2:**
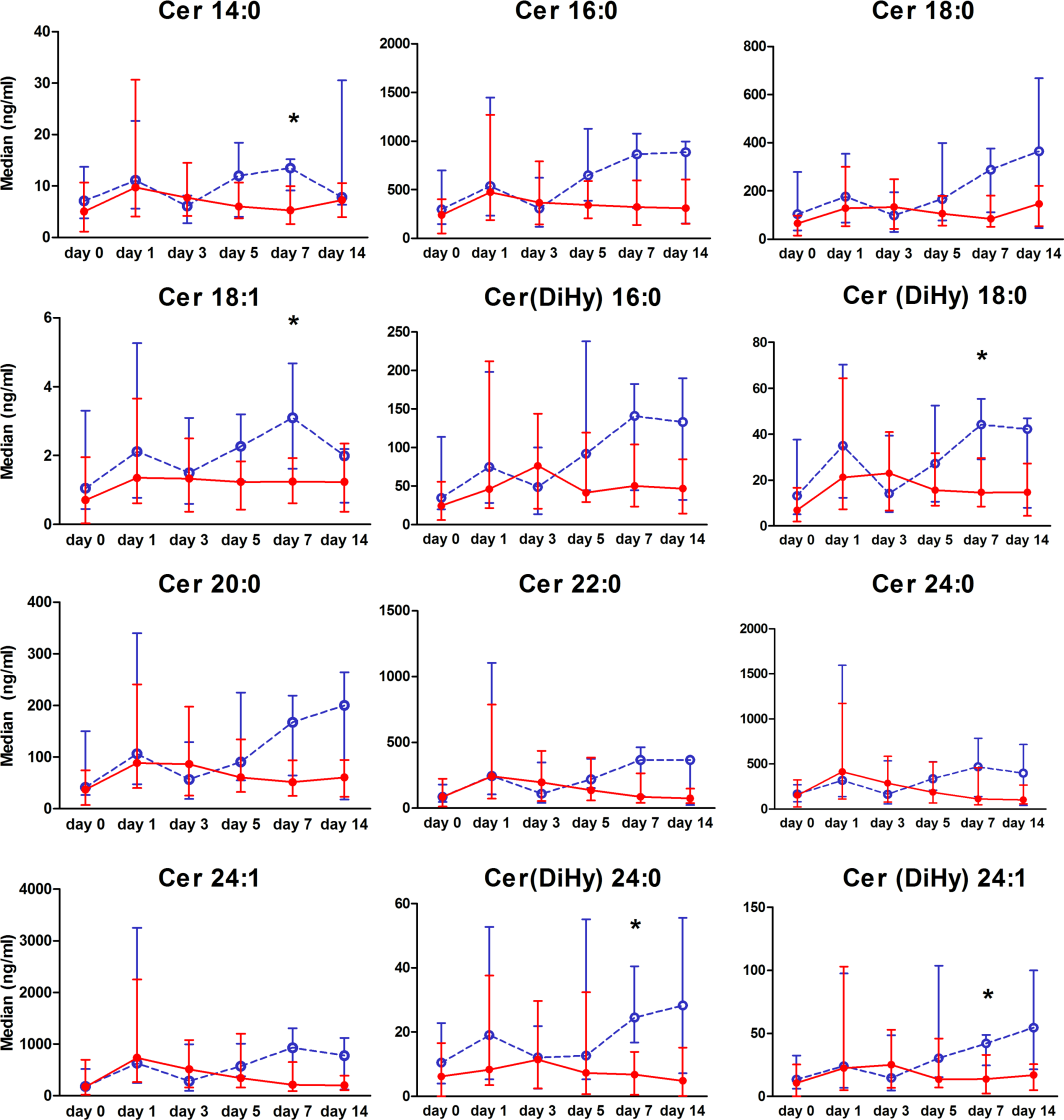
Ceramide patterns in preterm infants with and without bronchopulmonary dysplasia (BPD). Ceramide patterns (ng/ml) in tracheal aspirates for infants who developed BPD (BPD; ●, continuous line) and infants who did not developed BPD (○, dashed line). Dots represent median values, whiskers 25^th^ and 75^th^ percentiles, and group comparison by means of univariate analysis per time point, * = *p* < 0.01.

We collected 341 TAs from 111 preterm infants included in our final analysis, of which 84 TAs were collected at day 0, 87 at day 1, 52 at day 3, 47 at day 5, 39 at day 7, and 32 at day 14. All ceramides were highly correlated with each other. Secretory IgA levels showed little variation between the samples, indicative of a low variance in dilution between different patient samples. All infants showed an increase in ceramides after the first day of intubation. Ceramides in infants who did not develop BPD generally showed a late rise in ceramides until day 14, and this was not seen in those who developed BPD. Median ceramide concentrations (ng/ml) over time with 25^th^ and 75^th^ percentiles in infants who did and did not develop BPD are presented in Fig 2.

The linear mixed model with mean log-transformed ceramide concentration as dependent variable found an overall significant difference in ceramide profile between patients who did and did not develop BPD (*p* = 0.026) and a significant change in ceramide profile over time compared to the day of intubation (Table 2). Multivariable logistic regression analysis showed that the ceramide profile over time had no additional predictive value over known predictors (GA at birth, birth weight, total days invasive ventilation) for the outcome BPD (Table 3). When we performed separate multivariable logistic regression analysis for all the individual ceramides for each time point, individual ceramides had no significant predictive value over the known predictors (S1 Table).

**Table 2:** The change in overall ceramide profile.

	Ceramide profile level	
Predictor	Coefficient (95% CI)	*p* value
GA at birth	-0.044 (-0.200–0.112)	0.575
Birth weight (SDS score)	0.177 (-0.049–0.403)	0.123
Total days invasive ventilation	0.014 (-0.009–0.037)	0.221
Time between birth and intubation (days)	0.002 (-0.003–0.007)	0.442
***BPD diagnosis***		
No BPD	0^a^	
BPD or respiratory death	-0.797 (-1.497–0.098)	0.026^b^
***Post intubation day***		
Day 0	0^a^	
Day 1	1.019 (0.565–1.472)	<0.001^b^
Day 3	0.458 (-0.083–1.000)	0.097
Day 5	0.871 (0.303–1.440)	0.003^b^
Day 7	0.634 (0.023–1.244)	0.042^b^
Day 14	0.410 (-0.257–1.076)	0.227

This linear mixed model investigates the association between GA, birth weight (SDS score), total days invasive ventilation, time between birth and intubation, time of obtaining TA (post intubation day 0, 1, 3, 5, 7 or 14) and the occurrence of BPD and respiratory death on rates of change in ceramide profile.

^a^ No BPD and post intubation day 0 are used as reference category

^b^ p<0.05.

**Table 3:** Multivariable logistic regression analysis of BPD and respiratory death.

Independent variable	OR	95% CI	*p* value
**GA at birth**	0.558^a^	0.327–0.954	0.033
**Birth weight (SDS score)**	0.884	0.424–1.846	0.486
**Total days mechanical ventilation**	1.126^a^	1.002–1.265	0.046
**Time between birth and intubation**	0.868	0.566–1.332	0.518
**Intercept ceramide profile**	0.836	0.493–1.420	0.508
**Slope ceramide profile**	0.604	0.205–1.776	0.359

The dependent variable in this analysis is BPD outcome (0 = did not develop BPD, 1 = did develop BPD or died because of respiratory failure). GA at birth, birth weight SDS, total days of invasive ventilation and time between birth and intubation were included as independent variables in the logistic regression model. n = 79, Hosmer-Lemeshow test (p = 0.468)

^a^ p< 0.05.

### Cell count in tracheal aspirates

**Fig 3:**
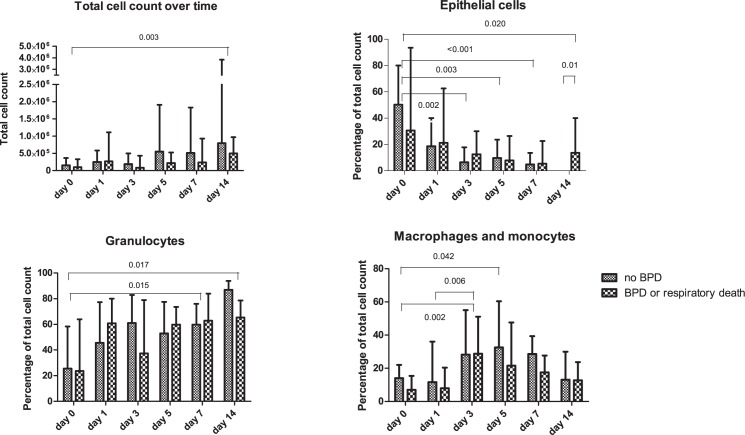
Cell distribution in TAs of preterm born infants. a) Total cell count for each time point between two groups (no BPD and BPD or respiratory death). b) Percentage of epithelial cells in TAs of infants who did not and who did develop BPD or died because of respiratory failure. c) Percentage of granulocytes in TAs of infants who did not and who did develop BPD or died because of respiratory failure. d) Percentage of macrophages or monocytes in TAs of infants who did not and who did develop BPD or died because of respiratory failure.

We found no difference in total cell count of TA samples between infants who did and who did not develop BPD or died because of respiratory failure. Overall, TAs acquired at day 14 contained significantly more cells than TAs obtained at day 0, adjusted for multiple comparisons; 530,000 (207,000–1,044,000) versus 136,500 (45,000–354,750), respectively, p = 0.003. Differential cell count showed a significant decrease over time of epithelial cells and a significant increase over time in granulocytes and macrophages and/or monocytes (Fig 3). Overall, there was a significant correlation between ceramide profile and total cell count, but no significant correlation between ceramide profile and percentage of granulocytes and macrophages or monocytes.

## Discussion

We showed an overall increase in ceramides in TAs of preterm infants after the first day of mechanical ventilation. Infants who developed BPD had lower ceramide concentrations compared to those who did not, in particular at day 7 after intubation. Ceramide profiles changed over time, and were significantly different between infants who did or did not develop BPD. However, the combined ceramide profiles over time had no additional predictive value over known predictors of BPD.

This is the first study investigating ceramide profiles in TAs of preterm born infants. We recently showed in a mouse model of BPD that changes in ceramide levels may be a factor in hyperoxia- induced lung injury, and affect proper lung development and function [19]. Also Kunzmann *et* al. showed an increase in ceramide levels in a sheep model of LPS-induced antenatal chorioamnionitis [20]. With this study we aimed to reproduce these findings in preterm infants. We collected TAs at multiple time points, which enabled us to observe the development of ceramide concentrations over time. The prospective design allowed us to investigate the predictive value of ceramide concentrations for BPD development when compared to known clinical risk factors.

Ceramides play an important role in apoptosis and lung inflammation [26] and mediate acute lung injury by increasing alveolar permeability and pro-inflammatory cytokine production [27]. Long chain ceramides (Cer 16:0, Cer 18:0 and Cer 20:0) have anti-proliferative and pro-apoptotic effects, whereas very long chain ceramides (Cer 22:0, Cer 24:0 and Cer 24:1) promote cell proliferation [28]. In the present study, we observed an early increase in both long chain and very long chain ceramides in TAs of preterm born infants after 1 day of mechanical ventilation and oxygen supplementation. This is consistent with our results obtained in the mouse model showing an early increase of ceramides after start of exposure to hyperoxia. In infants, we found a late increase of both long chain and very long chain ceramides in those who did not develop BPD. These results at first seem to be in contrast with our earlier findings of increased ceramides in hyperoxia-exposed mice [19]. However, in our study all preterm infants were exposed to both mechanical ventilation and supplemental oxygen. Therefore, we speculate that infants in our study were comparable to hyperoxia-exposed mice, as they showed an increase in ceramides directly after intubation and exposure to oxygen supplementation. Due to medical ethical reasons it was not possible to include a control group of infants who were not intubated. When we combined the individual ceramides into a ceramide profile we indeed found a significant change in the profile over time and a significant different profile in those infants who did develop BPD compared to the infants who did not develop BPD. In a multivariable analysis both the ceramide profile over time and the individual ceramides had no additional predictive value over GA at birth, birth weight, and total days mechanical ventilation. Altogether, an increased initial ceramide-triggered probably apoptotic signalling is present in all preterm infants exposed to mechanical ventilation and hyperoxia. Apoptosis of alveolar epithelial cells has been found before in the lungs of preterm infants that were subjected to ventilation and oxygen treatment [17] and in mouse models of BPD [29]. Second, our results suggest that a reduced late increase in ceramides may predispose preterm infants to develop BPD. We hypothesize that this late increase in ceramides suggests the need for both apoptosis and proliferation during relatively normal lung growth and development. Therefore, it is worthwhile to investigate the role of ventilation and hyperoxia-induced ceramide production in epithelial apoptosis as a mechanism responsible for pulmonary apoptosis and inhibition of alveolar development in preterm infants with BPD.

Snoek *et al*. investigated ceramide concentrations in infants born > 32 weeks GA with a congenital diaphragmatic hernia (CDH) and did not find a difference in ceramide concentrations between infants with and without BP [30]. This discrepancy may be explained by the fact that the pathophysiology of BPD development in neonates with CDH is different from that in preterm infants. Patients with CDH are not surfactant deficient [31] and inflammatory processes seem to play a less important role. It should be noted that the ceramide concentrations in TAs of infants with CDH were in the same range as in the present study. Therefore, lower ceramide concentrations might predispose for BPD both in infants with lung hypoplasia due to CDH and in ventilated preterm infants.

Ceramides can be increased by increased sphingomyelin metabolism via sphingomyelinases, by increased production via *de novo* synthesis or from sphingosine by ceramide synthase [5]. For this reason, it would be interesting to analyse the quantity of sphingomyelins in the TAs in our preterm infants. However, this was not feasible as most infants were treated with exogenous surfactant, which contains abundant sphingomyelins.

Pulmonary inflammation n has always been considered a hallmark of BPD development. Previous studies showed an association between increased pro-inflammatory cytokine concentrations in blood and lung derived fluid and development of BPD [32]. In the current study, we found a significant increase in granulocytes and macrophages or monocytes in TAs over time; however, this increase did not significantly differ between infants with or without later BPD. Furthermore, the overall ceramide profile did not correlate with the percentage of granulocytes and macrophages or monocytes, suggesting that the concentrations of ceramides might not be influenced by local inflammation in the lungs.

A few limitations of our study should be considered. We analysed ceramides in TAs obtained from routine suctioning procedures, and not in BAL fluid, which might yield larger samples and better standardization, but is more invasive. However, there is evidence that TAs are suitable substitutes for BAL samples in new-borns [33]. Second, it was impossible to obtain TAs from preterm born infants who did not need mechanical ventilation. Therefore, one can argue that a proper control group is missing. Few TAs (18%) were macroscopically contaminated with blood. Ceramides are present in plasma, and this might have influenced our results [34]. However, sensitivity analysis after excluding these specimens, yielded similar results. Patient characteristics between the infants who did and did not develop BPD differed with respect to GA at birth, birth weight, antenatal corticosteroids, postnatal surfactant, late onset sepsis, PDA and total days of mechanical ventilation. Based on a recent review regarding clinical predictors for BPD we chose only to correct for GA at birth, birth weight and total days of mechanical ventilation [35]. Correcting for all differentially expressed variables shown in Table 1 did not change the results, but yielded not enough statistical power due to the small sample size. The number of TA samples decreased over time, as many infants could be extubated within 14 days. This inevitably introduced selection bias. However, again sensitivity analysis including only those infants with at least one late TA sample available (13 infants without and 36 infants with BPD), yielded similar results. Also, one could argue about the fact that BPD development is a continuum and a binary division based on the 36 weeks definition will introduce loss of power. In the future, it will be interesting to relate ceramides to BPD severity based on for example structural lung abnormalities and lung function.

In conclusion, a pattern with early increase and subsequent decrease in ceramides in preterm infants exposed to mechanical ventilation and supplemental oxygen seems to predispose for BPD development. Therefore, ceramide profiles in TAs may be a new early marker for BPD and it is worthwhile to further investigate the role of ventilation- and hyperoxia induced ceramide production in pulmonary epithelium in preterm infants with BPD.

## Supporting information

S1 Data Database. (SAV)

S2 Data SPSS syntax. (DOCX)

S1 Table Multivariable logistic regression analysis of BPD or respiratory death. Multivariable logistic regression model corrected for GA at birth, birth weight (SDS score), total days of invasive ventilation and time between birth and intubation. Definition abbreviation: Cer = ceramide; Cer(DiHy) = dihydro-ceramide; OR = odds ratio; CI = confidence interval; p = p value. a p = 0.05, b not enough observations to perform logistic regression analysis.
(DOCX)
