# Supplementary material for: Ceramides in tracheal aspirates of preterm infants: Marker for bronchopulmonary dysplasia
Source: PLoS One. 2018 Jan 18;13(1):e0185969. doi: 10.1371/journal.pone.0185969 (PMC5773003; doi:10.1371/journal.pone.0185969)
Supplement: S2 Data — (DOCX) [file pone.0185969.s002.docx]

**S2 Data. SPSS syntax**

* PCA analysis

PCA.

USE ALL.

SPLIT FILE OFF.

* Based on Scree plot: 1 component extracted.

FACTOR

/VARIABLES LNCer14 LNCer16 LNCerDiHy16 LNCer18 LNCer18.1 LNCerDiHy18 LNCer20

LNCer22 LNCer24 LNCer24.1 LNCerDiHy24 LNCerDiHy24.1

/MISSING LISTWISE

/ANALYSIS LNCer14 LNCer16 LNCerDiHy16 LNCer18 LNCer18.1 LNCerDiHy18 LNCer20 LNCer22

LNCer24 LNCer24.1 LNCerDiHy24 LNCerDiHy24.1

/PRINT UNIVARIATE INITIAL CORRELATION KMO EXTRACTION ROTATION

/FORMAT SORT

/PLOT EIGEN ROTATION

/CRITERIA MINEIGEN(1) ITERATE(25)

/EXTRACTION PC

/CRITERIA ITERATE(25) DELTA(0)

/ROTATION OBLIMIN

/SAVE REG(ALL)

/METHOD=CORRELATION.

* Linear mixed model with main effects and random intercept (for each PCA factor) = with no BPD and day 0 as reference category = results in Table 3 of the article.

SPLIT FILE OFF.

MIXED PCA_1 BY Outcome_BPD2 time2 WITH GAdec sds_birthweight wk36i Time_betweendays

/CRITERIA=CIN(95) MXITER(100) MXSTEP(10) SCORING(1) SINGULAR(0.000000000001) HCONVERGE(0, ABSOLUTE) LCONVERGE(0, ABSOLUTE) PCONVERGE(0.000001, ABSOLUTE)

/FIXED= GAdec sds_birthweight wk36i Time_betweendays Outcome_BPD2 time2 | SSTYPE(3)

/METHOD=REML

/PRINT=SOLUTION TESTCOV

/RANDOM=INTERCEPT | SUBJECT(id) COVTYPE(VC)

/REPEATED=time2 | SUBJECT(id) COVTYPE(ID)

/SAVE=PRED RESID

/EMMEANS=TABLES(Outcome_BPD2) COMPARE ADJ(LSD).

* Multivariate logistic regression.

USE ALL.

SPLIT FILE OFF.

* Correlation matrix independent variables (GA, birthweight, sds_birthweight, Time_betweendays, sex, preeclampsia, chorioamnionitis, early and late sepsis, days invasive ventilation, days oxygen).

USE ALL.

COMPUTE filter_$=(time = 0).

VARIABLE LABELS filter_$ 'time = 0 (FILTER)'.

VALUE LABELS filter_$ 0 'Not Selected' 1 'Selected'.

FORMATS filter_$ (f1.0).

FILTER BY filter_$.

EXECUTE.

CORRELATIONS

/VARIABLES= Outcome_BPD gender GAdec birthweight sds_birthweight Time_betweendays preeclampsia PA_amnionitis Early_sepsis Late_sepsis

PDA_new wk36i wk36O2

/PRINT=TWOTAIL NOSIG

/MISSING=PAIRWISE.

* Multivariate logistic regression - Model 1a.

USE ALL.

LOGISTIC REGRESSION VARIABLES Outcome_BPD

/METHOD=ENTER ic_PCA1 sl_PCA1 GAdec sds_birthweight Time_betweendays

/PRINT=GOODFIT CI(95)

/CRITERIA=PIN(.05) POUT(.10) ITERATE(20) CUT(.5).

* Multivariate logistic regression - Model 1b (with other variables with significant differences between groups).

USE ALL.

LOGISTIC REGRESSION VARIABLES Outcome_BPD

/METHOD=ENTER ic_PCA1 sl_PCA1 GAdec sds_birthweight wk36i Time_betweendays prenatalsteroid

Surfactant_new Late_sepsis PDA_new

/CONTRAST (Surfactant_new)=Indicator(1)

/CONTRAST (Late_sepsis)=Indicator(1)

/CONTRAST (PDA_new)=Indicator(1)

/CONTRAST (prenatalsteroid)=Indicator(1)

/PRINT=GOODFIT

/CRITERIA=PIN(0.05) POUT(0.10) ITERATE(20) CUT(0.5).

* Multivariate logistic regression - Model 2a (average of log ceramide at each day).

USE ALL.

LOGISTIC REGRESSION VARIABLES Outcome_BPD

/METHOD=ENTER PCA_1.0 GAdec sds_birthweight Time_betweendays

/PRINT=GOODFIT CI(95)

/CRITERIA=PIN(.05) POUT(.10) ITERATE(20) CUT(.5).

LOGISTIC REGRESSION VARIABLES Outcome_BPD

/METHOD=ENTER PCA_1.1 GAdec sds_birthweight Time_betweendays

/PRINT=GOODFIT CI(95)

/CRITERIA=PIN(.05) POUT(.10) ITERATE(20) CUT(.5).

LOGISTIC REGRESSION VARIABLES Outcome_BPD

/METHOD=ENTER PCA_1.3 GAdec sds_birthweight Time_betweendays

/PRINT=GOODFIT CI(95)

/CRITERIA=PIN(.05) POUT(.10) ITERATE(20) CUT(.5).

LOGISTIC REGRESSION VARIABLES Outcome_BPD

/METHOD=ENTER PCA_1.5 GAdec sds_birthweight Time_betweendays

/PRINT=GOODFIT CI(95)

/CRITERIA=PIN(.05) POUT(.10) ITERATE(20) CUT(.5).

LOGISTIC REGRESSION VARIABLES Outcome_BPD

/METHOD=ENTER PCA_1.7 GAdec sds_birthweight Time_betweendays

/PRINT=GOODFIT CI(95)

/CRITERIA=PIN(.05) POUT(.10) ITERATE(20) CUT(.5).

LOGISTIC REGRESSION VARIABLES Outcome_BPD

/METHOD=ENTER PCA_1.14 GAdec sds_birthweight Time_betweendays

/PRINT=GOODFIT CI(95)

/CRITERIA=PIN(.05) POUT(.10) ITERATE(20) CUT(.5).

* Multivariate logistic regression - Model 2b (average of log ceramide at each day, with other variables with significant differences between groups).

USE ALL.

LOGISTIC REGRESSION VARIABLES Outcome_BPD

/METHOD=ENTER PCA_1.0 GAdec sds_birthweight wk36i Time_betweendays prenatalsteroid

Surfactant_new Late_sepsis PDA_new

/CONTRAST (Surfactant_new)=Indicator(1)

/CONTRAST (Late_sepsis)=Indicator(1)

/CONTRAST (PDA_new)=Indicator(1)

/CONTRAST (prenatalsteroid)=Indicator(1)

/PRINT=GOODFIT

/CRITERIA=PIN(0.05) POUT(0.10) ITERATE(20) CUT(0.5).

LOGISTIC REGRESSION VARIABLES Outcome_BPD

/METHOD=ENTER PCA_1.1 GAdec sds_birthweight wk36i Time_betweendays prenatalsteroid

Surfactant_new Late_sepsis PDA_new

/CONTRAST (Surfactant_new)=Indicator(1)

/CONTRAST (Late_sepsis)=Indicator(1)

/CONTRAST (PDA_new)=Indicator(1)

/CONTRAST (prenatalsteroid)=Indicator(1)

/PRINT=GOODFIT

/CRITERIA=PIN(0.05) POUT(0.10) ITERATE(20) CUT(0.5).

LOGISTIC REGRESSION VARIABLES Outcome_BPD

/METHOD=ENTER PCA_1.3 GAdec sds_birthweight wk36i Time_betweendays prenatalsteroid

Surfactant_new Late_sepsis PDA_new

/CONTRAST (Surfactant_new)=Indicator(1)

/CONTRAST (Late_sepsis)=Indicator(1)

/CONTRAST (PDA_new)=Indicator(1)

/CONTRAST (prenatalsteroid)=Indicator(1)

/PRINT=GOODFIT

/CRITERIA=PIN(0.05) POUT(0.10) ITERATE(20) CUT(0.5).

LOGISTIC REGRESSION VARIABLES Outcome_BPD

/METHOD=ENTER PCA_1.5 GAdec sds_birthweight wk36i Time_betweendays prenatalsteroid

Surfactant_new Late_sepsis PDA_new

/CONTRAST (Surfactant_new)=Indicator(1)

/CONTRAST (Late_sepsis)=Indicator(1)

/CONTRAST (PDA_new)=Indicator(1)

/CONTRAST (prenatalsteroid)=Indicator(1)

/PRINT=GOODFIT

/CRITERIA=PIN(0.05) POUT(0.10) ITERATE(20) CUT(0.5).

LOGISTIC REGRESSION VARIABLES Outcome_BPD

/METHOD=ENTER PCA_1.7 GAdec sds_birthweight wk36i Time_betweendays prenatalsteroid

Surfactant_new Late_sepsis PDA_new

/CONTRAST (Surfactant_new)=Indicator(1)

/CONTRAST (Late_sepsis)=Indicator(1)

/CONTRAST (PDA_new)=Indicator(1)

/CONTRAST (prenatalsteroid)=Indicator(1)

/PRINT=GOODFIT

/CRITERIA=PIN(0.05) POUT(0.10) ITERATE(20) CUT(0.5).

LOGISTIC REGRESSION VARIABLES Outcome_BPD

/METHOD=ENTER PCA_1.14 GAdec sds_birthweight wk36i Time_betweendays prenatalsteroid

Surfactant_new Late_sepsis PDA_new

/CONTRAST (Surfactant_new)=Indicator(1)

/CONTRAST (Late_sepsis)=Indicator(1)

/CONTRAST (PDA_new)=Indicator(1)

/CONTRAST (prenatalsteroid)=Indicator(1)

/PRINT=GOODFIT

/CRITERIA=PIN(0.05) POUT(0.10) ITERATE(20) CUT(0.5).
