# Supplementary material for: Ceramides in tracheal aspirates of preterm infants: Marker for bronchopulmonary dysplasia
Source: PLoS One. 2018 Jan 18;13(1):e0185969. doi: 10.1371/journal.pone.0185969 (PMC5773003; doi:10.1371/journal.pone.0185969)
Supplement: S1 Table — Multivariable logistic regression model corrected for GA at birth, birth weight (SDS score), total days of invasive ventilation and time between birth and intubation. Definition abbreviation: Cer = ceramide; Cer(DiHy) = dihydro-ceramide; OR = odds ratio; CI = confidence interval; p = p value. a p = 0.05, b not enough observations to perform logistic regression analysis. (DOCX) [file pone.0185969.s003.docx]

**Table S1. Multivariable logistic regression analysis of BPD or respiratory death**

|  | Day 0 | | | Day 1 | | | Day 3 | | | Day 5 | | | Day 7 | | |
| --- | --- | --- | --- | --- | --- | --- | --- | --- | --- | --- | --- | --- | --- | --- | --- |
| Independent variable | **OR** | **95% CI** | ***p*** | **OR** | **95% CI** | ***p*** | **OR** | **95% CI** | ***p*** | **OR** | **95% CI** | ***p*** | **OR** | **95%CI** | ***p*** |
| Cer 14:0 | 0.864 | 0.555-1.343 | 0.515 | 0.541 | 0.268-1.094 | 0.087 | 3.206 | 0.778-13.213 | 0.107 | 0.213 | 0.037-1.216 | 0.082 | 0.066 | 0.003-1.410 | 0.082 |
| Cer 16:0 | 0.858 | 0.564-1.305 | 0.474 | 0.520 | 0.264-1.027 | 0.060 | 2.517 | 0.759-8.346 | 0.131 | 0.319 | 0.089-1.150 | 0.081 | 0.210 | 0.030-1.472 | 0.116 |
| Cer 18:0 | 0.872 | 0.590-1.291 | 0.495 | 0.519 | 0.264-1.023 | 0.058 | 3.285 | 0.899-11.994 | 0.072 | 0.272 | 0.062-1.190 | 0.084 | 0.161 | 0.020-1.265 | 0.082 |
| Cer 18:1 | 0.986 | 0.628-1.547 | 0.950 | 0.837 | 0.544-1.290 | 0.421 | 1.540 | 0.702-3.377 | 0.281 | 0.373 | 0.122-1.141 | 0.084 | 0.013 | 0.000-1.340 | 0.066 |
| Cer(DiHy)16:0 | 0.835 | 0.581-1.200 | 0.331 | 0.615 | 0.328-1.153 | 0.130 | 1.769 | 0.724-4.323 | 0.211 | 0.419 | 0.143-1.229 | 0.113 | 0.171 | 0.025-1.185 | 0.074 |
| Cer(DiHy)18:0 | 0.957 | 0.679-1.350 | 0.803 | 0.849 | 0.537-1.342 | 0.484 | 1.721 | 0.755-3.921 | 0.196 | 0.355 | 0.109-1.156 | 0.086 | 0.092 | 0.008-1.102 | 0.060 |
| Cer 20:0 | 0.916 | 0.658-1.275 | 0.604 | 0.599 | 0.346-1.036 | 0.067 | 2.156 | 0.754-6.166 | 0.152 | 0.269 | 0.072-1.008 | 0.051 | 0.087^a^ | 0.007-1.043 | 0.054 |
| Cer 22:0 | 0.990 | 0.729-1.345 | 0.950 | 0.634 | 0.373-1.077 | 0.092 | 1.679 | 0.678-4.154 | 0.263 | 0.339 | 0.115-1.001 | 0.050 | 0.150 | 0.018-1.278 | 0.083 |
| Cer 24:0 | 0.939 | 0.716-1.230 | 0.646 | 0.628 | 0.372-1.060 | 0.081 | 1.493 | 0.644-3.465 | 0.350 | 0.374 | 0.138-1.014 | 0.053 | 0.087 | 0.005-1.401 | 0.085 |
| Cer 24:1 | 1.002 | 0.771-1.303 | 0.987 | 0.634 | 0.373-1.078 | 0.093 | 1.530 | 0.677-3.461 | 0.307 | 0.346 | 0.120-0.998 | 0.050 | 0.191 | 0.026-1.407 | 0.104 |
| Cer(DiHy)24:0 | 0.910 | 0.673-1.232 | 0.543 | 0.845 | 0.628-1.136 | 0.264 | 1.211 | 0.788-1.862 | 0.383 | 0.750 | 0.461-1.219 | 0.246 | ^a^ | ^a^ | ^a^ |
| Cer(DiHy)24:1 | 0.875 | 0.662-1.157 | 0.349 | 0.852 | 0.635-1.143 | 0.284 | 1.100 | 0.720-1.680 | 0.661 | 0.406 | 0.150-1.104 | 0.077 | 0.027 | 0.000-2.350 | 0.113 |
| Ceramide profile | 0.905 | 0.629-1.303 | 0.593 | 0.656 | 0.383-1.125 | 0.126 | 1.741 | 0.704-4.306 | 0.230 | 0.317 | 0.095-1.054 | 0.061 | 0.061 | 0.003-1.298 | 0.073 |

Multivariable logistic regression model corrected for GA at birth, birth weight (SDS score), total days of invasive ventilation and time between birth and intubation.

Definition abbreviation: Cer = ceramide; Cer(DiHy) = dihydro-ceramide; OR = odds ratio; CI= confidence interval; p = p value. Bonferroni correction, significance level p<0.01. ^a^ not enough observations to perform logistic regression analysis.
